# Reversible voltage dependent transition of abnormal and normal bipolar resistive switching

**DOI:** 10.1038/srep36953

**Published:** 2016-11-14

**Authors:** Guangyu Wang, Chen Li, Yan Chen, Yidong Xia, Di Wu, Qingyu Xu

**Affiliations:** 1 Department of Physics, Southeast University, Nanjing 211189, China; 2 National Laboratory of Solid State Microstructures, Nanjing University, Nanjing 210093 China; 3 Department of Materials Science and Engineering, Nanjing University, Nanjing 210008, China

## Abstract

Clear understanding the mechanism of resistive switching is the important prerequisite for the realization of high performance nonvolatile resistive random access memory. In this paper, binary metal oxide MoO_x_ layer sandwiched by ITO and Pt electrodes was taken as a model system, reversible transition of abnormal and normal bipolar resistive switching (BRS) in dependence on the maximum voltage was observed. At room temperature, below a critical maximum voltage of 2.6 V, butterfly shaped I-V curves of abnormal BRS has been observed with low resistance state (LRS) to high resistance state (HRS) transition in both polarities and always LRS at zero field. Above 2.6 V, normal BRS was observed, and HRS to LRS transition happened with increasing negative voltage applied. Temperature dependent I-V measurements showed that the critical maximum voltage increased with decreasing temperature, suggesting the thermal activated motion of oxygen vacancies. Abnormal BRS has been explained by the partial compensation of electric field from the induced dipoles opposite to the applied voltage, which has been demonstrated by the clear amplitude-voltage and phase-voltage hysteresis loops observed by piezoelectric force microscopy. The normal BRS was due to the barrier modification at Pt/MoO_x_ interface by the accumulation and depletion of oxygen vacancies.

There is a strong technological demand to develop faster, smaller, cheaper, and more reliable nonvolatile memory devices, which is therefore promoting the intensive researches on various mechanisms^1^,^2^. Among them, resistive random access memory (RRAM) has received much attention due to its simple structure, rapid operation, and high density integration^3^,^4^,^5^,^6^. In recent years, remarkable improvements have been made to understand the physics of RRAM devices^4^. Based on the polarity of applied voltages of switching, resistive switching (RS) characteristics between high resistance state (HRS) and low resistance state (LRS) can be classified into two types, i.e., unipolar and bipolar^4^,^7^,^8^. For unipolar resistive switching (URS), the set (HRS to LRS) and reset (LRS to HRS) processes happen at the same polarity. For the bipolar resistive switching (BRS), a resistance changing from HRS to LRS occurs at certain voltage polarity, with an inverse process from LRS to HRS with reversed voltage polarity. The mechanism has been intensively studied for the RS behavior. Based on the RS region, RS can be categorized into two types, bulk type with conducting filaments and interface type with tunneling barrier height and width modulation^7^,^9^,^10^,^11^. The reversible structure modification and resulted RS mainly originates from the thermal activated^7^ or electric field driven ion migration, such oxygen vacancies^12^,^13^, metallic electrode atoms^14^, etc. The RS is also very sensitive to the selected electrode, and abnormal BRS might be observed with fixed resistance state at zero field no matter the RS at high field^15^,^16^.

Oxides are the most intensively studied materials for RRAM. For the binary metallic oxide with RS based on migration of oxygen vacancies, those material with highly sensitive of resistivity on oxygen content is very attractive. It has been reported that molybdenum oxides show variable electrical conductivity from metallic MoO_2_ to insulating MoO_3_ with variation of oxygen content^17^,^18^, and amorphous MoO_x_ film shows 5 orders change of resistivity with oxygen partial pressure ranging from 1.00 × 10^−3^ mbar to 1.37 × 10^−3^ mbar^19^. In this paper, we report the RS in amorphous MoO_x_ films sandwiched by ITO and Pt electrodes. In contrast to the previous reports of abnormal BRS and normal BRS in distinctive structures^16^,^20^,^21^, reversible transition between abnormal BRS and normal BRS in dependence on the maximum applied voltage has been observed in the same structure. It has been suggested that the metastable pinning sites in the vicinity of equilibrium positions may immobilize the oxygen vacancies, leading to the induced dipoles with opposite electric field and abnormal BRS at low voltage. Higher voltage mobilizes the fixed oxygen vacancies, modify the barrier at Pt/MoO_x_, leading to the normal BRS.

## Results and Discussion

**Figure 1:**
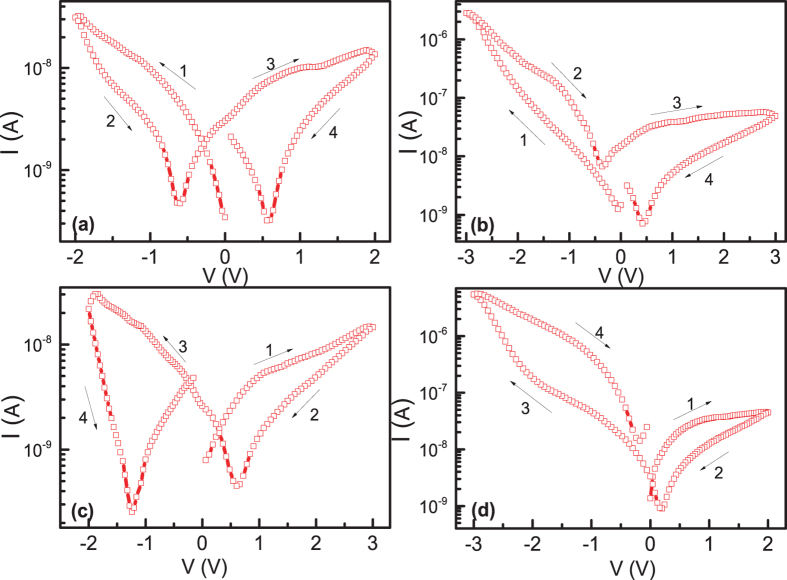
The I-V curves in semi-log scale of the Pt/MoO_x_/ITO device under different maximum voltages.

The sweeping voltage was applied on the bottom ITO electrode in the way of 0 → −2 → 0 → 2 → 0 V (shown in the Fig. 1(a) in semi-log scale). During the measurements, no current compliance was applied. In this paper, we take the absolute value of current, and the original I-V curves in linear scale are shown in Figure S1. It should be noted that we do not need a power consuming electroforming process to realize the resistive switching, which is usually required in conventional filament-like resistive switching^22^. The fresh memory cells of Pt/MoO_x_/ITO sandwiched structure were always in the LRS state, and gradually switched to the HRS state with increasing voltage in both polarities. Interestingly, the cell switched back to the LRS state at zero field, which is a typical abnormal BRS^16^. In contrast to the general observed abnormal BRS with negligible current at zero field^16^,^23^,^24^, significantly large current can be observed for our cells at zero bias, and the I-V curves showed a butterfly shape with current minimum at voltage of −0.6 V and 0.56 V after the voltage switching back from the maximum value. The significant large current at zero voltage clearly suggested the existence of some internal electric field inside the cell depending on the voltage polarization. Similar phenomenon has also been observed in other films, e.g. SrTiO_3_^25^, BiFeO_3_^26^. With increasing the maximum voltage to 3 V applied in the same sequence as Fig. 1(a), HRS to LRS switching was observed in the negative voltage branch, LRS state was preserved at zero voltage and switched back to HRS with increasing positive voltage (shown in Fig. 1(b)). The cell can keep the LRS and HRS states stably depending on the voltage polarity, which is a typical normal BRS. If we decreased the maximum voltage to 2 V again, the similar abnormal BRS behavior as Fig. 1(a) can be recovered (shown in Figure S2). Thus, in the same cell, we can reversibly switch between abnormal BRS and normal BRS by the maximum voltage. We further checked whether the HRS to LRS switching can happen in the positive voltage branch if we applied positive voltage to 3 V first, only LRS to HRS switching can be observed, as shown in Fig. 1(c). It should be noted that we even first applied positive voltage to 9 V, but only LRS to HRS switching was observed (Figure S3). If we applied the positive voltage first, and then applied negative voltage to maximum value of −3 V, clear HRS to LRS switching can be observed, and normal BRS was observed again (Fig. 1(d)). From above results, we can conclude that abnormal to normal RS can be observed in the negative voltage branch in dependence on the maximum voltage (LRS to HRS with low maximum voltage, and HRS to LRS with high maximum voltage), while in positive voltage branch only LRS to HRS switching can be observed.

**Figure 2:**
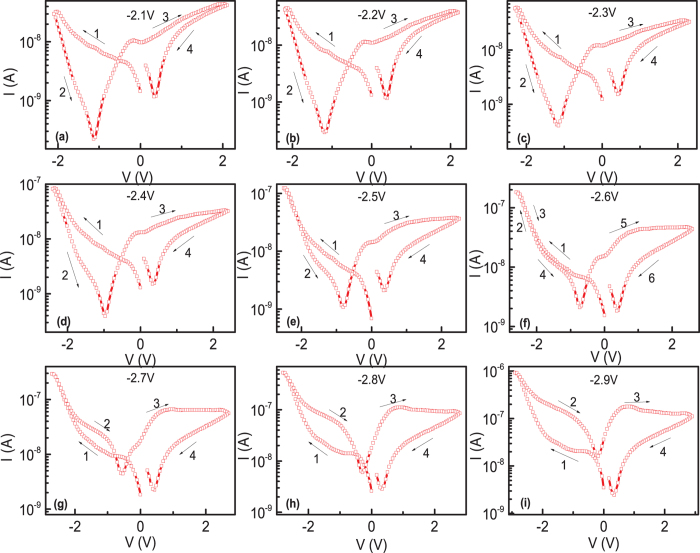
(**a**–**i**) The I-V curves in semi-log scale with increasing the maximum voltage in the step of 0.1 V.

To clear understand the evolution of abnormal BRS to normal BRS, the maximum voltage was gradually increased in a range of 2.1 to 2.9 V in step of 0.1 V, and the corresponding I-V curves are shown in Fig. 2. As can be seen, the clear abnormal BRS behavior can be observed with butterfly shaped I-V curves when small maximum voltage was applied. With increasing the maximum voltage, the current value of HRS state in step 2 became larger. There is a critical maximum voltage (V_C_) of 2.6 V, at which the LRS curve in step 1 and HRS curve in step 2 are nearly overlapping. With further increasing the maximum voltage above 2.6 V, the LRS and HRS interchanged. The resistance state in step 1 now changed to HRS state, and a HRS to LRS switching happened in the negative voltage branch. The separation between the current value in the curves of step 1 and 2 became larger with increasing the maximum voltage, and normal BRS can be observed, which persisted with further increasing the maximum voltage, as shown in Figure S4 (maximum voltage of 5 V).

**Figure 3:**
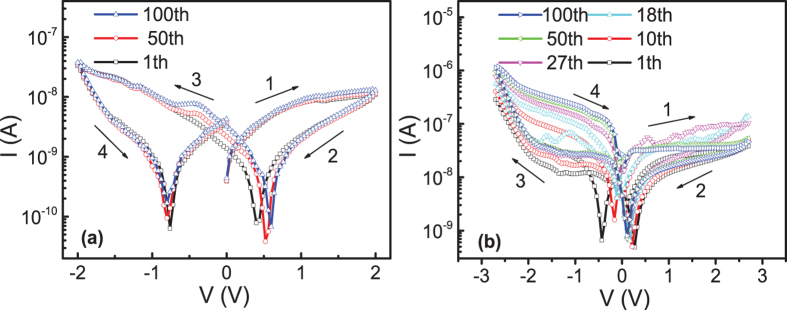
(**a**,**b**) Show the consecutive 100 switching cycles in maximum voltage of 2.0 V and 2.7 V respectively.

The stability property of BRS in the Pt/MoO_x_/ITO device below and above the V_C_ at room temperature was measured, and is shown in Fig. 3. When we applied the maximum voltage of 2 V, it shows that the consecutive 100 switching cycles almost overlap with each other, which indicates a stable abnormal BRS behavior, as can be seen in Fig. 3(a). However, if we applied the maximum voltage (2.7 V) to just above the V_C_ of 2.6 V, a gradual evolution of the consecutive I-V curves can be observed, as can be seen in Fig. 3(b). In the initial I-V curve, the separation between HRS and LRS in the negative branch is small. After the consecutive I-V measurements, both the currents at HRS and LRS increased with broader separation between the HRS and LRS branches. It can be seen that the increase of current value in LRS state is much faster than that in the HRS state. Less significant shift can be observed in the positive branch. Furthermore, it can be seen that the I-V curves became nearly stable after the consecutive 50 runs. This can be understood by the effect of electric field on the migration of oxygen vacancies. Under high voltage, the electric field can drag the oxygen vacancies out from the equilibrium positions. However, the electric field is not strong enough when the maximum voltage is just above V_C_, and it will take time for the migration of oxygen vacancies. The final state needs time to reach due to the low migration speed of oxygen vacancies, leading the gradual evolution of I-V curves. The time dependent HRS and LRS in normal BRS was further measured, and shown in Figure S5, which showed the highly stable retention.

**Figure 4:**
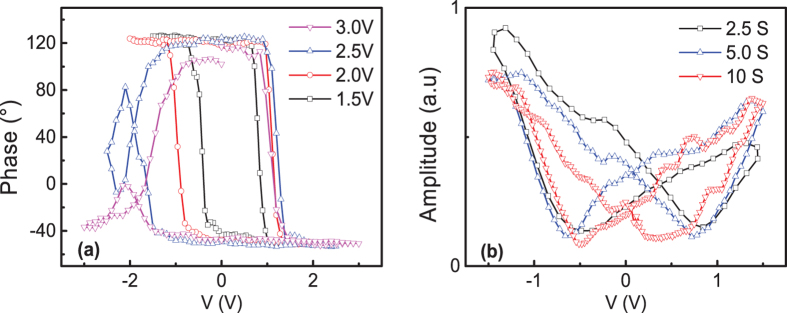
Out-of-plane (**a**) PFM phase-voltage and (**b**) amplitude-voltage curves for Pt/MoO_x_/ITO sandwiched structure with various maximum voltages and periods.

In contrast to the previous reported I-V curves with current minimum nearly overlapping at zero voltage, butterfly-shaped I-V curves were observed with two current minimums in the Pt/MoO_x_/ITO structure, which is quite similar to the slow dielectric relaxation of induced dipoles in ferroelectric materials^26^. The significant large current at zero voltage clearly demonstrated the existence of internal electric field. We performed the piezoelectric force microscopy (PFM) measurements, the amplitude-voltage and phase-voltage curves are shown in Fig. 4. The clear observation of the hysteretic butterfly-shaped loops is the general indication of ferroelectricity. However, Q. N. Chen *et al*. have observed the similar phenomenon in soda-lime glass which is clearly not ferroelectric and they attributed this to the induced dipole moment^27^. For the induced dipole moment, the phase-voltage loops showed significant broadening with increasing maximum voltage and variation of the shape of amplitude-voltage loops with increasing measuring periods, while nearly unchanged shape for ferroelectric materials^27^. As can be seen, the phase-voltage loops were significantly broadened with increasing maximum voltage and the shape of amplitude-voltage loops varied notably with increasing the measuring period. With the maximum voltage above 2.5 V (close to V_C_), the phase-voltage loops became distorted. The phase-voltage curves became more distorted with larger maximum voltages, as shown in Figure S6. Thus, the abnormal BRS can be understood by the induced dipoles due to the shift of oxygen vacancies from the equilibrium positions to the metastable sites, which induced an electric field opposite to the applied electric field, leading to the switching from LRS to HRS.

**Figure 5:**
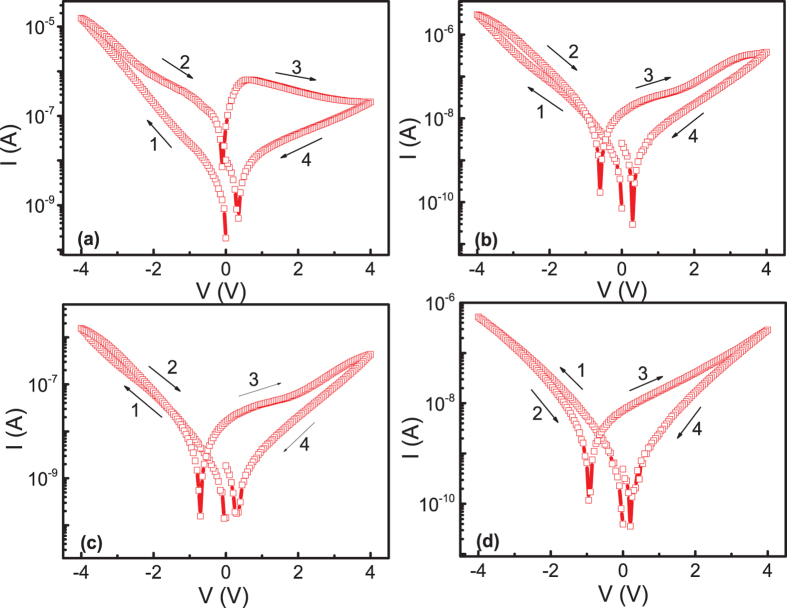
The I-V curves in semi-log scale at different temperatures with maximum sweeping voltage of 4 V. (**a**) 300 K; (**b**) 270 K; (**c**) 260 K. (**d**) 240 K.

Temperature always influences the migration rate of oxygen vacancies. We also measured the temperature dependent RS of our device in the step of 5 °C with decreasing temperature from 300 K. Figure 5 shows the RS behavior with maximum voltage of 4 V at various temperatures. As can be seen, stable normal BRS can be observed at 300 K. However, the HRS and LRS branches in I-V curves at the negative voltage branches became overlapped if we decreased the temperature to 270 K. The abnormal BRS became more obvious if we further decreased the temperature, and finally stable abnormal BRS can be observed at temperature of 240 K. This can be understood by the thermal activated migration of oxygen vacancies. With decreasing temperature, higher electric field was needed to drag the oxygen vacancies from the metastable sites to migrate inside the films. The average activation energy values vary from 0.139–0.262 eV (comparable to the known value of 0.141 eV (at 325 K))^28^ for different constant voltages from 1 V to 5 V (Figure S7). It is clearly revealed that the migration of oxygen vacancies is significantly decreased by the temperature.

**Figure 6:**
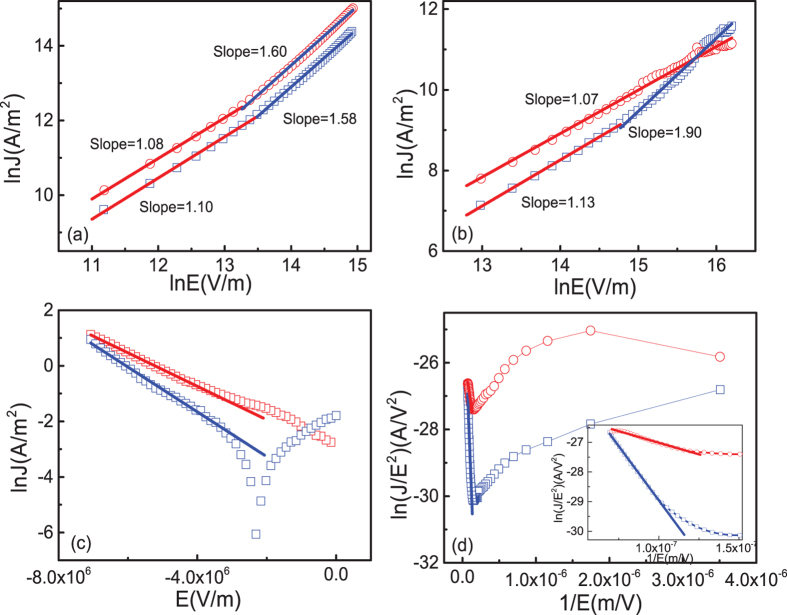
Typical I-V curves of Pt/MoO_x_/ITO devices plotted by different fittings for abnormal BRS in (**a**) positive (ohmic and SCLC) and (**c**) negative (electron tunneling) branches, and normal BRS in (**b**) positive (ohmic and SCLC) and (**d**) negative (FN tunneling) branches.

Several models have been attempted to interpret the RS phenomenon in RRAM^28^,^29^,^30^,^31^,^32^,^33^,^34^. In order to identify which model is suitable to describe the behavior in our devices, we analyzed the I-V curves of the BRS behavior by various models, such as space charge limited current (SCLC)^29^,^32^ Schottky emission^30^,^33^ Poole-Frenkel (PF) emission^28^,^34^ and electron tunneling^35^. Figure 6 shows the best fitting of the typical I-V curves of LRS and HRS in voltage branches of both abnormal and normal BRS. For abnormal BRS, Ohmic and SCLC are more obvious in the positive voltage (Fig. 6(a)), and in the negative voltage, LnI is proportional to V (Fig. 6(c))^35^, indicating the electron tunneling conduction because the migration of oxygen vacancies by electric field is negligible. For the positive branches in normal behavior, as can be seen, the dependence of LnJ and LnE for the LRS shows nearly linear relation with slope close to 1, indicating the Ohmic conduction, while the slope of the curve changes from 1.13 to 1.90, showing the typical trap-controlled SCLC mechanism (Fig. 6(b))^29^,^32^. In the negative branches in normal BRS, to investigate the dominant conduction mechanism in the junction, we used several conduction mechanisms to fit the I−V curves, including Schottky emission, PF emission, SCLC, and Fowler−Nordheim (FN) tunneling (Figure S8). The dielectric constants obtained by the fitting using Schottky emission or PF emission significantly deviate from the ideal value, excluding these two mechanisms (Figure S8). The good linear fitting at high voltage region between LnJ/E^2^ and 1/E suggests that FN tunneling is the dominant mechanism (Fig. 6(d)). To clarify which interface played the main role in the RS, we replaced the top Pt electrodes by Ti electrodes. As can be seen in Figure S9, the resistance became at least one order smaller, and only negligible RS can be observed. This confirms that RS happened at the Pt/MoO_x_ interface. The conduction mechanism in negative branches change from abnormal BRS to normal BRS can be understood by the different Pt/MoO_x_ interface state. In the abnormal BRS, the oxygen vacancies are fixed close to the equilibrium position, and will not modify the sharp interface between the Pt and MoO_x_, making it susceptible to electron tunneling^16^. In the normal BRS, the Pt/MoO_x_ interface was modified by the migration of oxygen vacancies under enough high electric field. Due to the accumulation of oxygen vacancies at Pt/MoO_x_ interface, the interface layer became thicker. The electrons can tunnel through the potential barrier at high electric field due to the formation of the triangle potential barrier^36^.

**Figure 7:**
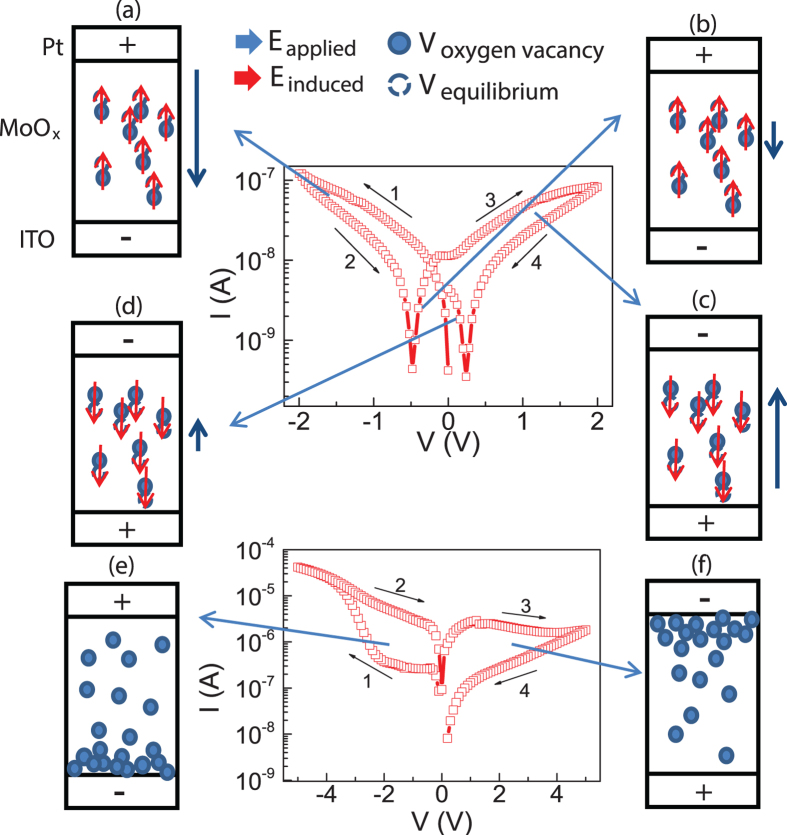
The schematic diagram of RS mechanism of Pt/MoO_x_/ITO sandwiched structure for abnormal BRS (**a**–**d**), and normal BRS (**e**,**f**).

The Fermi level of the MoO_x_ (4.89 eV)^37^ is close to the ITO work function (4.7 eV)^38^ but much lower than Pt (5.65 eV). Thus, the ITO/MoO_x_ interface tends to be Ohmic contact, and the RS mainly happens at the Pt/MoO_x_ interface. In order to assist us to better understand the microscopic mechanism of this BRS, a schematic diagram under different maximum applied electric fields is drawn and shown in Fig. 7. As can be seen, under small voltage, the oxygen vacancies cannot migrate inside the film, but slightly shift from the equilibrium positions to the metastable positions. The dipoles are formed, which induce the electric field opposite to the applied field. Due to the compensation of the external electric field, the actual internal electric field is smaller, leading to the higher resistance (Fig. 7(a)). With decreasing the voltage in the negative branch to some value, the electric field from dipoles is larger than the external applied electric field due to the much slow dielectric relaxation^26^, current is reversed and the cell gradually switches back to LRS, as the oxygen vacancies shift back to the equilibrium positions (Fig. 7(b)). With further increasing positive voltage, similar process will happen with only the polarity is reversed (Fig. 7(c,d)). When a higher maximum voltage is applied (>2.6 V at room temperature), oxygen vacancies will not be fixed around the equilibrium positions, and can migrate inside the film. When a positive voltage was applied, oxygen vacancies would move toward the Pt/MoO_x_ interface and accumulate there (Fig. 7(f)), leading to the higher trap density and the transition from LRS to HRS. When the voltage is reversed, the oxygen vacancies drift away from the interface (Fig. 7(e)), leading to the decrease of trap density. Under high electric field, triangle potential barrier is formed at the interface layer and electrons can tunnel through this barrier layer, leading to the HRS to LRS switching.

## Conclusion

In summary, a reversible transition between abnormal BRS and normal BRS has been observed in Pt/MoO_x_/ITO sandwiched structures without electroforming process. The critical maximum voltage for the transition between abnormal BRS and normal BRS is 2.6 V at room temperature, which increases with decreasing temperature. Under low maximum voltage, the electric field is not large enough to drive the oxygen vacancies, which can only occupy the metastable sites near the equilibrium positions. The dipoles were formed, inducing the electric field which may partially compensate the external applied field. Thus LRS to HRS transition can always be observed in both polarities, leading to the abnormal BRS. Under high maximum voltage, the oxygen vacancies can migrate inside the film, the accumulation/depletion of oxygen vacancies at Pt/MoO_x_ interface will modulate the trap density, leading the normal BRS.

## Methods

Amorphous MoO_x_ films were prepared by pulsed laser deposition (PLD) on indium-tin-oxide (ITO) substrates under various oxygen pressures from 0.2 Pa to 5.0 Pa at room temperature. The amorphous nature of the films was confirmed by X ray diffraction (XRD), as shown in Figure S10(a). The thickness of MoO_x_ films were estimated to be 277 nm from cross-sectional sample observed by a field emission scanning electron microscope (FE-SEM), as shown in Figure S11(a). Then 100 nm thick Pt top electrodes with diameter of 160 μm were sputtered on the MoO_x_ films through a shadow mask. The I–V characteristics of the Pt/MoO_x_/ITO sandwich structures were measured by a Keithley 2400 SourceMeter and 2182 A Nanovoltmeter, as the schematic structure shown in Figure S11(b). As can be seen from Figure S12, the resistivity of MoO_x_ film increased dramatically with increasing oxygen partial pressure above 1.0 Pa. In this paper, we selected the MoO_x_ film prepared under oxygen partial pressure of 4.0 Pa for the electrical characterization, which exhibited the most significant RS behavior. The valence state of Mo was characterized by X ray photoelectron spectroscopy (XPS, ThermoFisher SCIENTIFIC) with Al Kα X-ray source (h*ν* = 1486.6 eV), shown in Figure S10(b) and Figure S10(c), which confirms the existence of oxygen vacancies. The I-V curves at different temperatures were measured by a Cascade Summit 11000 M. The piezoelectric hysteresis loops were characterized by a scanning probe microscope (SPM, Asylum Research Cypher).

## Additional Information

**How to cite this article**: Wang, G. *et al*. Reversible voltage dependent transition of abnormal and normal bipolar resistive switching. *Sci. Rep.* **6**, 36953; doi: 10.1038/srep36953 (2016).

**Publisher’s note**: Springer Nature remains neutral with regard to jurisdictional claims in published maps and institutional affiliations.

## Supplementary Material

Supplementary Information
