# Supplementary material for: Reversible voltage dependent transition of abnormal and normal bipolar resistive switching
Source: Sci Rep. 2016 Nov 14;6:36953. doi: 10.1038/srep36953 (PMC5107888; doi:10.1038/srep36953)
Supplement: Supplementary Information [file srep36953-s1.docx]

**Supplemental material**

Reversible voltage dependent transition of abnormal and normal bipolar resistive switching

Guangyu Wang,^1^ Chen Li,^2,3^ Yan Chen,^2,3^ Yidong Xia,^2,3^ Di Wu,^2,3^ & Qingyu Xu^1,2,*^

**Figure S1** The linear curves of the Pt/MoO_x_/ITO device under different maximum voltages. It should be noted here that the current takes the initial value (and the absolute value elsewhere in the paper.). The insets of (b) and (d) show the local enlarged view.

^1^Department of Physics, Southeast University, Nanjing 211189, China. ^2^National Laboratory of Solid State Microstructures, Nanjing University, Nanjing 210093 China. ^3^Department of Materials Science and Engineering, Nanjing University, Nanjing 210008, China.

* Corresponding author: xuqingyu@seu.edu.cn

**Figure S2** The similar abnormal BRS behavior can be recovered when we decreased the maximum voltage to 2 V again.

**Figure S3** The I-V curves in semi-log scale of the Pt/MoOx/ITO sandwiched device measured under maximum positive voltage to (a) 9 V and (b) 5 V.

**Figure S4** Normal BRS can be observed with further increasing the maximum voltage to 5 V.

**Figure S5** The retention properties of MoO_x_ sample measured at -0.5 V after being switched to HRS and LRS states in the normal BRS process.

**Figure S6** Out-of plane PFM phase hysteresis loop for Pt/MoO_x_/ITO with increasing maximum voltage from 3 V to 5 V.

**Figure S7** Ln σ versus 1000/T plots for the MoO_3_ thin films.

The activation energy (E_a_) can be calculated by the Arrhenius equation,

$$\sigma=\sigma_{0}\exp\left(\frac{-E_{a}}{K_{B}T}\right)\cdot\cdot\cdot\cdot\cdot\cdot\text{（}1\text{）}$$

(whereσis the conductivity,σ_0_ is a constant, E_a_ is the activation energy, K_B_ is the Boltzmann constant and T is the absolute temperature). The average activation energy values vary from 0.139-0.262 eV for different constant voltages from 1 V to 5 V, comparable to the reported value of 0.141eV (at 325K).^1^

To investigate the dominant conduction mechanism, we used several conduction mechanisms to fit the J−V curves, including Schottky emission, Poole-Frenkel (PF) emission, SCLC, and Fowler−Nordheim (FN) tunneling. These mechanisms can be described by the following equations:

$$J_{S}=A\text{*}T^{2}\text{exp}\left[\frac{-q\left(\varphi_{B}-\sqrt{qE/4\pi\varepsilon_{\text{r}}\varepsilon_{0}}\right)}{k_{B}T}\right]\cdot\cdot\cdot\cdot\cdot\cdot\text{（}2\text{）}$$

$$J_{PF}=qN_{c}\mu E\text{exp}\left[\frac{-q\varphi_{t}}{k_{B}T}+\frac{q\sqrt{qE/\pi\varepsilon_{r}\varepsilon_{0}}}{rk_{B}T}\right]\cdot\cdot\cdot\cdot\cdot\cdot\text{（}3\text{）}$$

$$J_{SCLC}=\frac{9\mu\varepsilon_{0}\varepsilon_{\text{r}}}{8d}E^{2}\cdot\cdot\cdot\cdot\cdot\cdot\cdot\cdot\cdot\cdot\cdot\cdot\cdot\cdot\cdot\cdot\cdot\cdot\cdot\cdot\cdot\cdot\cdot\cdot\cdot\cdot\cdot\cdot\cdot\cdot\cdot\cdot\cdot\text{（}4\text{）}$$

$$J_{FN}=CE^{2}\text{exp}\left(-\frac{D^{2}\sqrt{\phi_{i}^{3}}}{E}\right)\cdot\cdot\cdot\cdot\cdot\cdot\cdot\cdot\cdot\cdot\cdot\cdot\cdot\cdot\cdot\cdot\cdot\cdot\cdot\cdot\cdot\cdot\cdot\text{（}5\text{）}$$

where ε_r_ is the relative dielectric constant, ε_0_ the permittivity of free space, K_B_ the Boltzmann constant; N_c_ the density of charge carriers in the conduction band, r the coefficient ranged between 1 and 2, d the film thickness,μ the charge carrier mobility, A, and C the constants, ϕ, and φ_i_ the height of Schottky barrier and potential barrier height, respectively.

Through fitting the J−E curves in ln(J/T^2^) − E^1/2^ (Schottky), ln(J/E) − E^1/2^ (PF), log(J) − log(E) (SCLC or Ohmic) and ln(J/E^2^) − 1/E (FN) relations, the mechanism that dominants the conduction process could be determined. Figure S8 (a) and (b) show the curves in Schottky emission and PF emission. The linear fittings are good in the high voltage regions, however, the dielectric constants derived from the Schottky and PF emissions are 0.2728 and 0.1409, 1.5736 and 0.7864, respectively, which significantly deviate the ideal value (the value is 8),^2^ indicating that the Schottky and PF emissions are not reasonable in our devices. The slopes of the SCLC are 3.44 and 7.98 (Figure S8(c)), significantly deviate from the ideal value of 2. An interface-limited FN tunneling is used to fit the J-E curves at high electric fields in negative direction (Figure S8(d)). The measured I-V curves are fitted with straight lines, confirming the FN tunneling mechanism. So we can conclude that FN tunneling is the dominant mechanism in normal BRS at high fields in negative branches.

**Figure S8** Fitting results used to identify (a) Schottky emission, (b) PF emission, (c) SCLC, and (d) FN tunneling and (inset) a local enlarged view.

**Figure S9** I-V curves in semi-log scale of the similar cell structure of Ti/MoO_x_/ITO with only replacing the Pt electrode by Ti electrode.

**Figure S10** (a) XRD patterns of amorphous MoO_x_ thin film, only the diffraction peaks from ITO can be observed (marked by “*”). (b) XPS spectra of the Mo 3d_5/2_ and Mo 3d_3/2_, which locate at 232.4 eV and 236.4 eV, respectively, indicating the mainly +6 valence state of Mo.^3^ (c) The O 1s XPS spectrum, which was fitted by two peaks O_a_ and O_b_, respectively. The low binding energy component located at 530.1 eV (O_a_) is originated from the lattice oxygen in MoO_3_.^4^ The peak centered at 531.2 eV (O_b_), is assigned to the oxygen vacancies in the matrix of oxides.^5,6^ Thus, the XPS results confirm the existence of oxygen vacancies in MoO_x_ film.


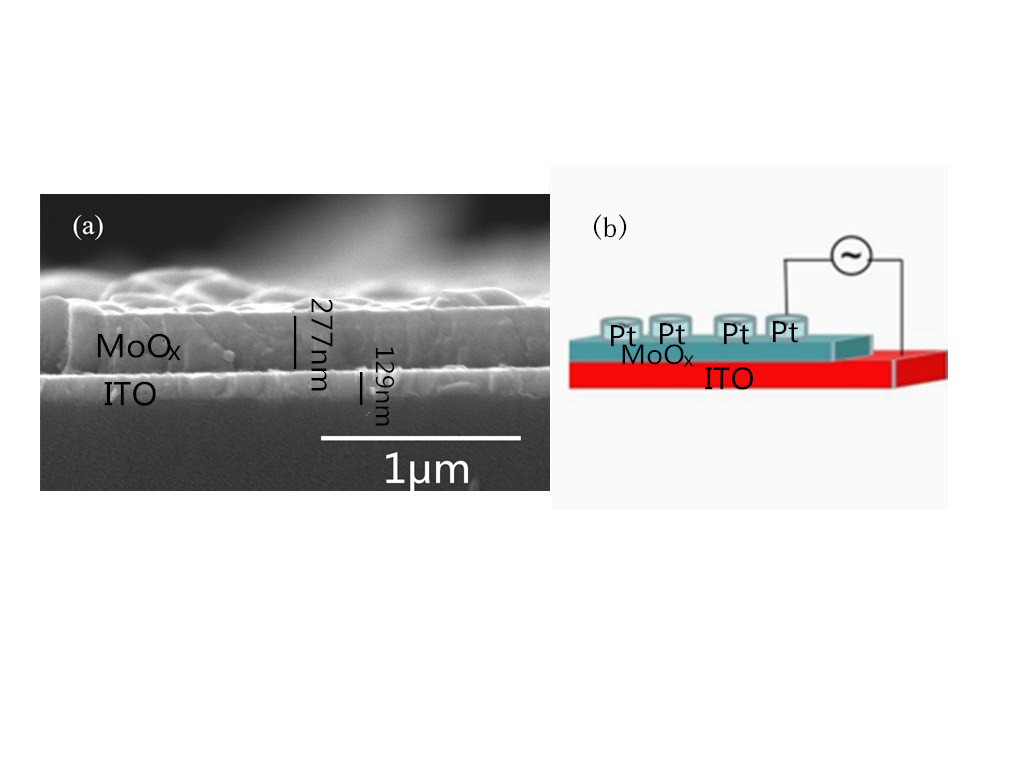


**Figure S11** (a) Cross-sectional FE-SEM image of MoO_x_ film, (b) the schematic diagram of the Pt/MoO_x_/ITO sandwiched structure.

**Figure S12** The resistivities of MoO_x_ with increasing oxygen partial pressure measured with in-plane geometry, the resistance of the film prepared under oxygen pressure of more than 3.5 Pa was too high to be measured. The resistive switching behavior has been studied for all the samples, and the film prepared under O2 pressure of 4.0 Pa shows the best performance.

**References**

1. Miyata, N., Suzuki, T., Ohyama, R., Physical properties of evaporated molybdenum oxide

films, *Thin Solid Films* 281, 218–222 (1996).

2 Vidya, S.; Solomon, S.; Thomas, J. K. Synthesis and Characterisation of MoO3 and WO3 Nanorods for Low Temperature Co-Fired Ceramic and Optical Applications. *J. Mater. Sci. Mater. Electron*. **26**, 3243–3255 (2015).

3 Greiner, M. T.; Helander, M. G.; Wang, Z. B.; Tang, W. M.; Qiu, J.; Lu, Z. H. A Metallic Molybdenum Suboxide Buffer Layer for Organic Electronic Devices. *Appl. Phys. Lett.* **96**, 213302 (2010).

4 Khyzhun, O. Y.; Strunskus, T.; Solonin, Y. M. XES, XPS and NEXAFS Studies of the Electronic Structure of Cubic MoO_1.9_ and H_1.63_MoO_3_ Thick Films. *J. Alloys Compd.* **366** (1–2), 54–60 (2004).

5 Chen, M.; Wang, X.; Yu, Y. H.; Pei, Z. L.; Bai, X. D.; Sun, C.; Huang, R. F.; Wen, L. S. X-Ray Photoelectron Spectroscopy and Auger Electron Spectroscopy Studies of Al-Doped ZnO Films. *Appl. Surf. Sci.* **158**, 134–140 (2000).

6 Yang, G.; Gao, D.; Shi, Z.; Zhang, Z.; Zhang, J.; Zhang, J.; Xue, D. Room Temperature Ferromagnetism in Vacuum-Annealed CoO Nanospheres. *J. Phys. Chem. C* **114**, 21989–21993 (2010).
